# Effect of analgesia nociception index monitor-based nociception control on perioperative stress responses during laparoscopic surgery in Trendelenburg position: a randomized controlled trial

**DOI:** 10.3389/fmed.2023.1196153

**Published:** 2023-08-04

**Authors:** Seung Hyun Kim, Chul Ho Chang, Jeong-Rim Lee, Seok Kyo Seo, Young In Kwon, Jae Hoon Lee

**Affiliations:** ^1^Department of Anesthesiology and Pain Medicine, Anesthesia and Pain Research Institute, Yonsei University College of Medicine, Seoul, Republic of Korea; ^2^Department of Obstetrics and Gynecology, Institute of Women’s Life Medical Science, Yonsei University College of Medicine, Seoul, Republic of Korea; ^3^Department of Anesthesiology and Pain Medicine, Yonsei University College of Medicine, Seoul, Republic of Korea

**Keywords:** analgesia, catecholamine, glucocorticoid, laparoscopy, nociception

## Abstract

**Introduction:**

The analgesia nociception index (ANI) monitor is a nociception monitoring device based on heart rate variability. We aimed to determine the effect of ANI monitor-based intraoperative nociception control on the perioperative stress response during laparoscopic surgery in the Trendelenburg position.

**Methods:**

Altogether, 72 female patients who underwent total laparoscopic hysterectomy were randomized to either the control or ANI group. Intraoperative nociception was controlled by remifentanil administration in a conventional manner (based on blood pressure and heart rate) in the control group and by ANI monitoring in the ANI group. Perioperative stress responses were estimated by measuring the levels of serum catecholamines and catabolic stress hormones at three timepoints: after loss of consciousness, at the end of surgery, and 1 h after the end of surgery.

**Results:**

The serum cortisol level at the end of surgery was significantly higher in the ANI group than in the control group (*p* < 0.001), although more remifentanil was administered in the ANI group than in the control group (*p* < 0.001). Changes in the other estimators’ levels were comparable between groups during the perioperative period. The hemodynamic profiles during surgery were also significantly different between the two groups. Phenylephrine use to treat hypotension was more common in the ANI group than in the control group (*p* = 0.005). However, postoperative clinical outcomes such as pain and nausea/vomiting did not differ between groups.

**Conclusion:**

ANI monitor-based nociception control in laparoscopic surgery in the Trendelenburg position did not improve perioperative stress responses, intraoperative opioid consumption, or postoperative clinical outcomes.

**Clinical trial registration:** ClinicalTrials.gov (NCT04343638).

## 1. Introduction

Adequate pain control is important for enhanced recovery after surgery. Although laparoscopy has improved postoperative clinical outcomes (1, 2), such as early mobilization and hospital discharge in gynecologic abdominal surgeries, patients undergoing total laparoscopic hysterectomy have been reported to experience severe pain after surgery (3).

Pain in conscious patients is assessed based on the patient’s self-reported pain scores such as numerical rating scale (NRS); however, the pain scores cannot be used to monitor nociception in unconscious patients. Conventionally, anesthesiologists control nociception according to indirect indicators of pain such as patient movement, high blood pressure, and tachycardia during surgery (4). However, these are not objective indicators of nociception, and nociception cannot be excluded even in the absence of these clinical symptoms.

Recently, several objective monitoring devices for nociception have been developed and applied in clinical practice (5). The analgesia nociception index (ANI) monitor (MetroDoloris, Lille, France) is a nociception monitoring device that non-invasively quantifies a patient’s nociception based on heart rate variability. Heart rate variability can be affected by various factors such as the autonomic system, body temperature, baroreflex, and neuroendocrine system. However, high-frequency heart rate variability >0.15 Hz specifically reflects parasympathetic nerves (6), and the ANI monitor calculates heart rate variability mediated by parasympathetic changes. Nociceptive stimulation reduces parasympathetic action and consequently decreases the ANI score. The ANI score ranges from 0–100, with 100 indicating maximum parasympathetic action and the lowest level of nociception and 0 indicating minimum parasympathetic action and the highest level of nociception. According to the manufacturer’s recommendation, the target level for adequate nociceptive control is between 50 and 70 of the average ANI value measured over the previous 240 s (ANIm) (7).

CO_2_ pneumoperitoneum induces hemodynamic changes, such as decreased cardiac output, increased systemic and pulmonary vascular resistance, increased arterial pressure, and activation of the sympathetic nervous system. Cardiac output decreases with increasing insufflation pressure (8), and vascular resistance increases immediately after pneumoperitoneum, which is related to neurohormonal factors (9). The steep Trendelenburg position, which is needed in laparoscopic pelvic surgeries, increases central blood volume and activates the baroreflex to decrease sympathetic nerve activity (10). Therefore, pneumoperitoneum and the steep Trendelenburg position in laparoscopic hysterectomy may disturb ANI monitoring because they can affect autonomic nervous system activity.

The previous studies on ANI monitor-based nociception control have reported inconsistent pain outcomes (4, 11–17), and this study aimed to evaluate the effectiveness of ANI monitoring-based nociception control in laparoscopic surgery in the steep Trendelenburg position. Although there is no definitive method to assess nociception during surgery, nociceptive stimulation during surgery can induce various neuroendocrine stress responses, resulting in increased catecholamines and catabolic hormones such as cortisol and adrenocorticotropic hormone (18–21). Therefore, we investigated whether ANI monitor-based nociception control can reduce the perioperative stress response compared with conventional nociception control by comparing serum catecholamines, stress hormones, and inflammatory cytokines in patients undergoing total laparoscopic hysterectomy.

## 2. Materials and methods

The Institutional Review Board of Yonsei University Health System, Seoul, South Korea, approved this trial (#4–2020-0130) on April 1, 2020. This study was registered before patient enrolment at ClinicalTrial.gov (NCT04343638, Principal investigator: Jae Hoon Lee, Date of registration: April 13, 2020) and conducted between May 2020 and October 2021. All participants provided written informed consent. Female patients aged 20–65 years who underwent elective total laparoscopic hysterectomy for uterine myoma or adenomyosis were recruited. We excluded patients with ASA physical status class ≥IV, endometriosis, anteroposterior diameter of uterus >12 cm, cognitive disorders, cardiac arrhythmia, implantable pacemaker, chronic opioid use, diseases affecting the autoimmune system (such as immune disease or diabetic neuropathy), use of medications affecting ANI monitoring (antimuscarinics, alpha-agonists, beta blockers), illiteracy, or foreigners. A computer-generated random code generator was used to randomly allocate participants to either the control group (conventional nociception control group) or ANI group (ANI monitor-guided nociception control group) in a 1:1 ratio. The patients and investigators in charge of data collection and outcome analyses were blinded, but the anesthesia providers were not blinded to randomization.

### 2.1. Surgery, anesthesia, and nociception management

On arrival in the operating room, standard monitoring, including pulse oximetry, non-invasive blood pressure monitoring, electrocardiography, and bispectral index (BIS) monitoring, was performed in all patients. ANI monitoring was also applied to all participants. While nociception control was performed based on ANI monitoring in the ANI group, the ANI monitor was covered with black paper so that it could not be observed in the control group. For the induction of general anesthesia, 2 mg/kg of propofol was administered, and remifentanil was infused using an effect-site target-controlled infusion pump with an initial target effect-site concentration of 3 ng/mL according to the Minto model. After confirmation of loss of consciousness, 0.6 mg/kg of rocuronium was administered. After endotracheal intubation, the patients were mechanically ventilated with 6–8 mL/kg predicted body weight of tidal volume, and respiratory rates were adjusted to maintain the end-tidal CO_2_ between 35 and 40 mmHg. Anesthetic depth was maintained with sevoflurane to adjust the BIS value between 40 and 60 in all patients throughout the surgery. According to the attending anesthesiologist’s decision, an additional 10 mg of rocuronium was administered to maintain a train-of-four count of less than 2 throughout the surgery.

In the control group, the remifentanil concentration was adjusted to 0.5 or 1.0, according to the judgment of the attending anesthesiologist based on mean arterial pressure (MAP) and heart rate. In the ANI group, when the ANIm values deviated from the target value of 50–70 (7), the concentration of remifentanil was adjusted by 0.5 or 1.0. Remifentanil concentration lower than 1.5 ng/mL was not allowed in either group.

Total laparoscopic hysterectomies were performed with two ports in the 30° Trendelenburg position by four expert gynecologists. Pneumoperitoneum was maintained with an intra-abdominal pressure of 12 mmHg and a CO_2_ flow rate of 20 L/min during the surgery. The specimens were extracted transvaginally in specimen bag.

All patients received 8 mg ondansetron 30 min before the end of surgery. In addition, 1 g acetaminophen was administered to patients weighing >50 kg, and 15 mg/kg acetaminophen was administered to patients weighing <50 kg 30 min before the end of surgery.

### 2.2. Hemodynamic management

Phenylephrine was used to correct isolated hypotension (MAP <60 mmHg) without bradycardia. Combined hypotension and bradycardia (<45 bpm) was managed with ephedrine. Glycopyrrolate was administered to treat the isolated bradycardia. In the case of bradycardia refractory to glycopyrrolate, atropine was used.

### 2.3. Post-anesthetic care unit

On arrival at the post-anesthetic care unit (PACU), NRSs for pain and nausea were assessed. Oxycodone (0.05 mg/kg) was used when the NRS score for pain was higher than 4, and 10 mg metoclopramide was infused when the NRS score for nausea was higher than 4. Patients were discharged from the PACU after an NRS for pain lower than 4 was reported.

### 2.4. ANI value analysis

ANI data recorded at 1-s intervals were downloaded after discontinuation of the main anesthetic at the end of surgery. The mean ANIm values from the start of induction of general anesthesia to cessation of an anesthetics, percentage of time with adequate analgesia with an ANIm value of 50–70, and percentage of time with inadequate analgesia with an ANIm value <50 were assessed for each patient.

### 2.5. Outcome measures

The primary outcomes in the present study were perioperative levels of catecholamines, stress hormones, and inflammatory cytokines. Blood sampling was performed at three timepoints: after loss of consciousness, at the end of surgery, and 1 h after the end of surgery. A peripheral venous catheter for blood sampling was inserted immediately after loss of consciousness and maintained until the final sampling. Blood samples were centrifuged and stored at −80°C until analysis. Quantification of serum catecholamines (norepinephrine, epinephrine), stress hormones (cortisol, adrenocorticotropic hormone), and inflammatory cytokines (interleukin-6, interleukin-10, and high mobility group box 1) was performed using enzyme-linked immunosorbent assay kits.

Secondary outcomes included intraoperative MAP and heart rate, duration of surgery, total dose of remifentanil and phenylephrine usage, ephedrine and anticholinergic use, NRS for pain at the PACU at five timepoints (at admission and 10, 20, 30, and 40 min after admission), NRS score for nausea at PACU admission and 30 min after PACU admission, usage of oxycodone and metoclopramide in the PACU, and duration of PACU stay.

### 2.6. Statistical analyses

Previously, the mean (standard deviation) of serum norepinephrine levels in patients undergoing laparoscopic gynecologic surgery at the end of surgery was reported to be 750 (320) pg./mL (22). Assuming that a 30% reduction in norepinephrine level is clinically meaningful, and considering a 10% dropout rate, 72 patients were required to provide 80% power at a significance level of 0.05.

Descriptive data are presented as the mean (standard deviation) or median (interquartile range). For between-group comparisons, Student’s t-test or Mann–Whitney U-test was used for continuous variables, and the chi-squared test or Fisher’s exact test was used for categorical variables. We performed repeated analyses of variance with group, time, and the interaction between group and time for repeated variables (catecholamine and stress hormone levels, mean arterial pressure, heart rate, and postoperative pain scores). All statistical analyses were performed using SPSS Statistics for Windows version 23 (IBM, Armonk, NY).

## 3. Results

**Figure 1:**
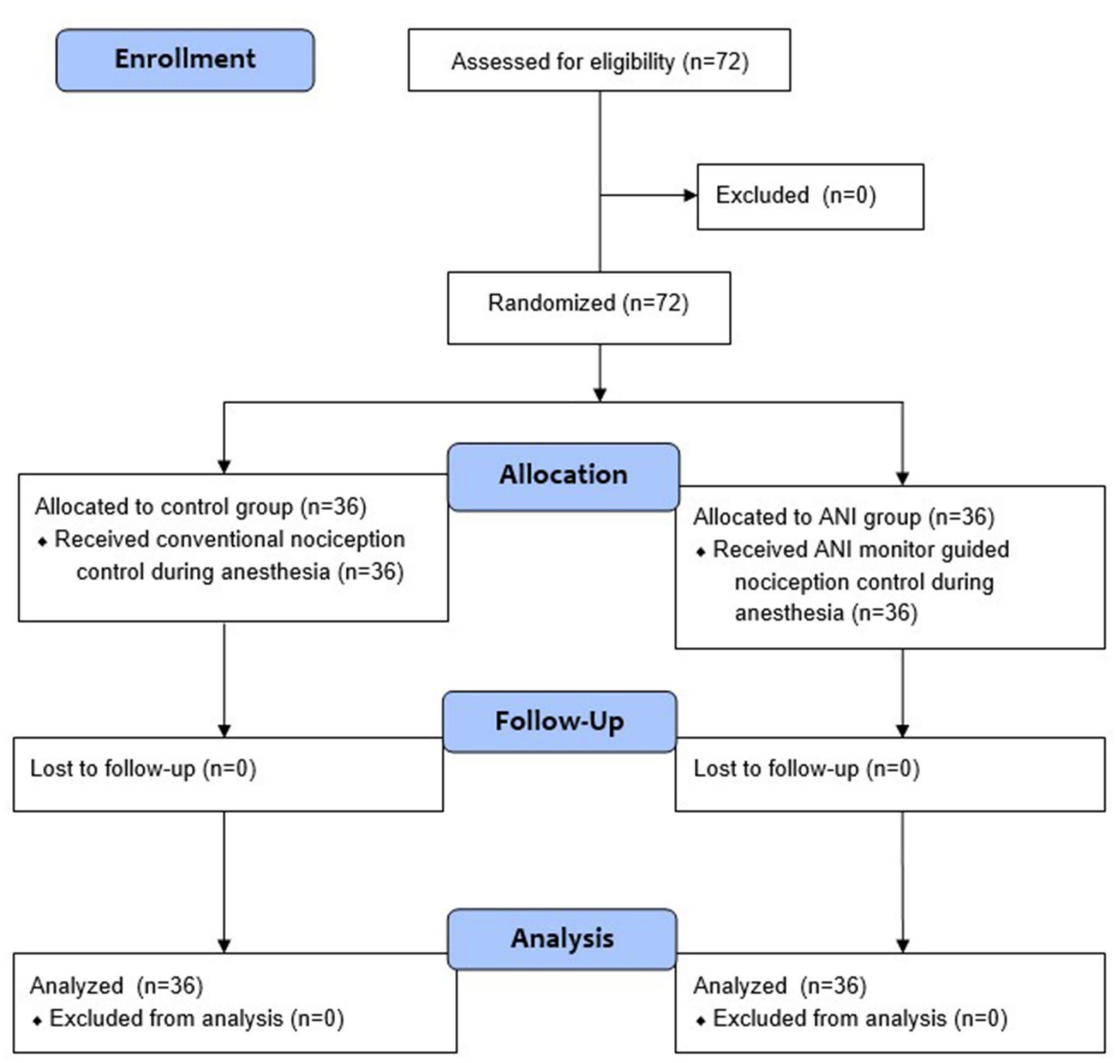
CONSORT flowchart.

A total of 72 patients deemed eligible for this study were randomly allocated to either the control group or the ANI group (Figure 1). The baseline patient demographics are presented in Table 1. Table 2 shows the ANI values and remifentanil administration data. There was no significant difference in the mean ANIm values during anesthesia and surgery. The percentage of time spent with adequate ANIm values between 50 and 70 was higher in the ANI group than in the control group (48.9 [9.2] % vs. 40.8 [13.9] %, *p* = 0.005). However, during surgery, the percentage of time spent with adequate ANIm values did not differ between the two groups. Remifentanil consumption was significantly higher in the ANI group than in the control group.

**Table 1:** Baseline patient characteristics.

	Control group (*N* = 36)	ANI group (*N* = 36)
Age (years)	47.0 (42.3, 49.0)	46.5 (44.0, 48.8)
Height (cm)	159.5 (156.8, 163.0)	160.1 (156.3, 165.0)
Weight (kg)	57.0 (53.3, 64.8)	59.5 (53.0, 65.8)
BMI (kg.m^−2^)	22.7 (20.8, 25.2)	22.9 (20.8, 25.9)
ASA class 1/2/3 [N(%)]	18 (50.0)/14 (38.9)/4 (11.1)	15 (41.7)/18 (50.0)/3 (8.3)
Indication (myoma, adenomyosis, coexistence of myoma and adenomyosis) [*N*(%)]	25 (77.8)/ 5 (13.9)/ 3 (8.3)	25 (69.4)/ 5 (13.9)/ 6 (16.7)
Medication [*N*(%)]		
None	25 (69.4)	26 (72.2)
^†^Cardiovascular	4 (11.1)	4 (11.1)
Metabolic	3 (8.3)	2 (5.6)
Others	8 (22.2)	6 (16.7)

Data are presented as median (interquartile range) for continuous variables and count (percentage) for categorical variables. ANI, analgesia nociception index.

^†^Cardiovascular, antihypertensive, and anti-arrhythmic medication except beta-blockers.

**Table 2:** Intraoperative data of ANI values and remifentanil consumption between the groups.

	Control group (*N* = 36)	ANI group (*N* = 36)	*p*-value
ANIm values			
Total	59.4 (7.6)	59.0 (6.8)	0.804
Before surgery	52.0 (9.1)	55.6 (7.2)	0.067
During surgery	61.3 (8.5)	59.9 (7.6)	0.451
Percentage of time with ANIm between 50 and 70			
Total	40.8 (13.9)	48.9 (9.2)	0.005^†^
Before surgery	31.7 (15.1)	43.6 (16.8)	0.002^†^
During surgery	43.8 (16.7)	50.4 (11.1)	0.055
Percentage of time with ANI <50			
Total	32.3 (16.4)	28.2 (14.7)	0.147
Before surgery	51.4 (23.6)	38.3 (17.2)	0.009^†^
During surgery	26.7 (17.8)	25.4 (17.0)	0.362
Remifentanil consumption (μg.kg^−1^.m^−1^)	0.052 (0.044, 0.060)	0.171 (0.126, 0.230)	<0.001^†^

Data are presented as mean (SD). ANI, analgesia nociception index; ANIm, average analgesia nociception index value measured over the previous 240 s. ^†^*p* < 0.05, compared with the control group.

**Figure 2:**
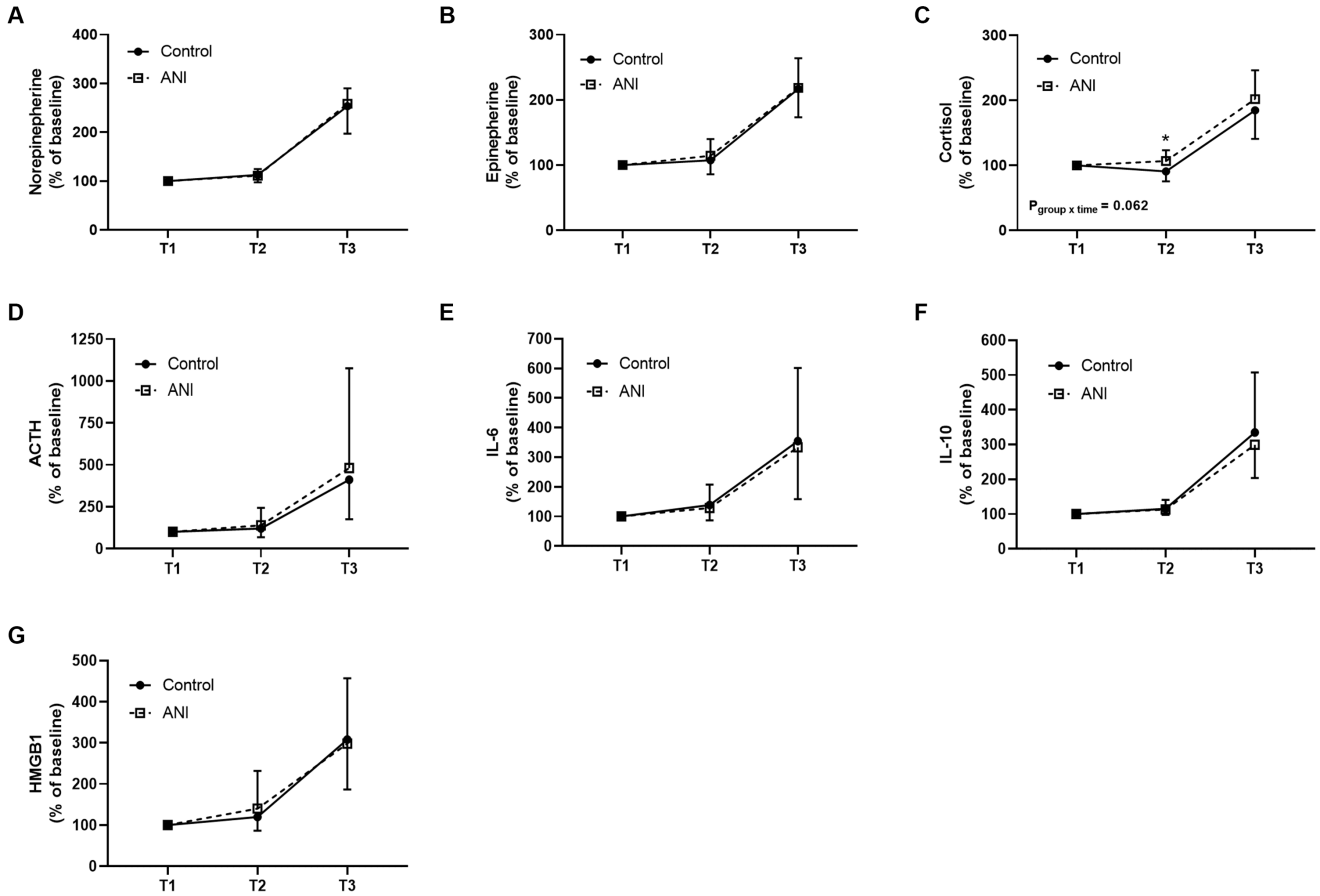
Perioperative changes in serum **(A)** norepinephrine, **(B)** epinephrine, **(C)** cortisol, **(D)** adrenocorticotropic hormone, **(E)** interleukin-6, **(F)** interleukin-10, and **(G)** high mobility group box 1 of the control and ANI group at three timepoints. T1, after the loss of consciousness; T2, at the end of surgery; T3, 1 h after the end of surgery; ANI, analgesia nociception index.

Figure 2 depicts the changes in biochemical markers reflecting the stress response during surgery or perioperative inflammation and tissue damage. Although the changes in cortisol levels in the perioperative period were not significantly different between the two groups (Figure 2C *p*_group × time_ = 0.062), the cortisol level at the end of surgery (T2) was significantly higher in the ANI group than in the control group in the post-hoc analysis (*p* < 0.001). The changes in other markers were comparable between the two groups.

**Figure 3:**
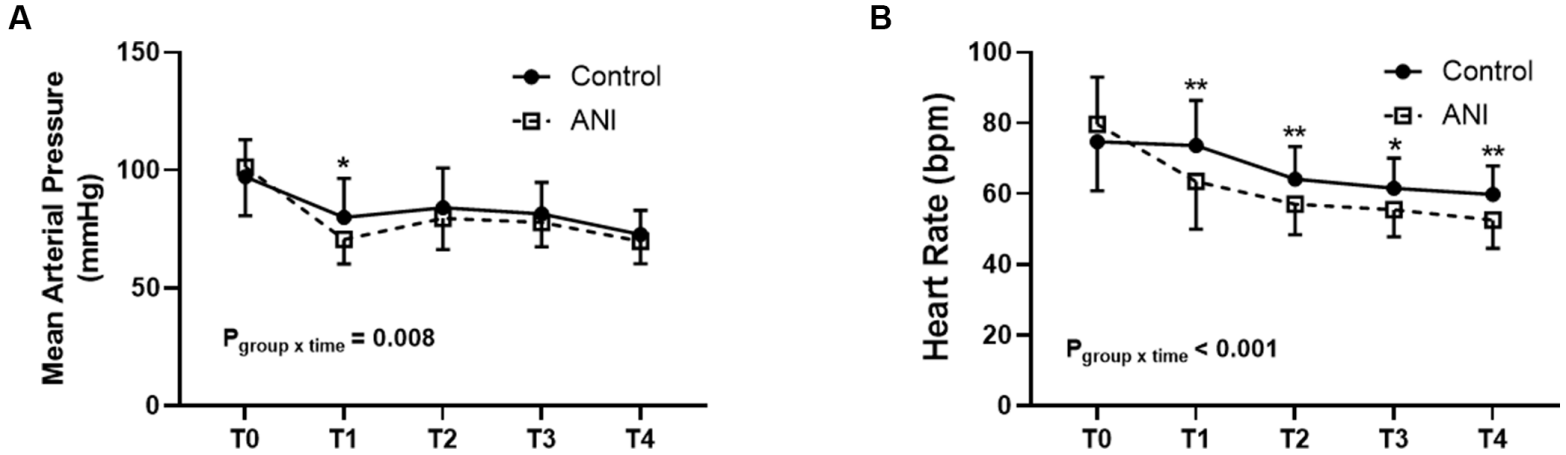
Intraoperative changes of mean arterial pressure **(A)** and heart rate **(B)** between the control and ANI group. T0, before induction of anesthesia; T1, after induction of anesthesia; T2, 10 min after the start of surgery; T3, 30 min after the start of surgery; T4, at the end of surgery. *p < 0.05; **p < 0.01. ANI, analgesia nociception index.

Other intraoperative and postoperative data are summarized in Table 3. Although the duration of surgery was similar between groups, a larger amount of phenylephrine was used in the ANI group than in the control group. Anticholinergics were used only in the ANI group. The change in MAP was different between the two groups (*p*_group × time_ = 0.008, Figure 3A), and MAP immediately after anesthetic induction was significantly lower in the ANI group than in the control group. Heart rate was also lower in the ANI group than in the control group from anesthetic induction until the end of surgery (*p*_group × time_ < 0.001, Figure 3B).

**Table 3:** Other intraoperative and postoperative data.

	Control group (*N* = 36)	ANI group (*N* = 36)	*p*-value
Intraoperative data			
Duration of surgery (min)	97.5 (74.0, 120.8)	94.0 (84.0, 117.8)	0.261
Fluid intake (ml)	700.0 (512.5, 800.0)	700.0 (700.0, 837.5)	0.175
Urine output (ml)	100.0 (50.0, 150.0)	100.0 (70.0, 150.0)	0.340
Blood loss (ml)	65.0 (50.0, 150.0)	65.0 (50.0, 100.0)	0.496
Phenylephrine use, N (%) of patients	11 (30.6)	23 (63.9)	0.005^†^
Phenylephrine dose (μg.min^−1^)	0.0 (0.0, 9.8)	20.9 (0.0, 34.7)	0.001^†^
Ephedrine use, N (%) of patients	3 (8.3)	3 (8.3)	>0.999
Anticholinergics use, N (%) of patients	0 (0.0)	5 (13.9)	0.054
Data in post-anesthesia care unit			
Duration of PACU stay (min)	60 (55, 70)	60 (51, 73)	0.501
Number of oxycodone administration	2 (1, 2.75)	2 (1, 2)	0.153
Nausea score at PACU admission	0.0 (0.0, 5.0)	0.0 (0.0, 3.8)	0.307
Nausea score at 30 min after PACU admission	1.0 (0.0, 4.0)	0.0 (0.0, 4.0)	0.327
Metoclopramide use, *N* (%) of patients	14 (38.9)	12 (33.3)	0.624

Data are presented as median (IQR) for continuous variables and count (percentage) for categorical variables. ANI, analgesia nociception index; PACU, post-anesthetic care unit. ^†^*p* < 0.05, compared with the control group.

Pain scores during PACU stay did not differ between the two groups (*p*_group × time_ = 0.255; Supplementary Figure 1). As shown in Table 3, oxycodone use did not differ between groups. Other parameters related to nausea and vomiting did not significantly differ between groups.

## 4. Discussion

ANI monitoring has been approved and adopted in clinical practice in many countries; however, the effect of ANI monitor-based nociception control during surgery on clinical outcomes has not been fully determined. The current study demonstrated that the application of ANI in laparoscopic hysterectomy did not reduce perioperative catecholamine levels when compared with conventional nociception control using hemodynamic variables. Furthermore, the serum cortisol level at the end of surgery was higher in the ANI group than in the control group, despite the higher dose of intraoperative remifentanil used. Therefore, the conventional application of ANI monitoring cannot improve the perioperative stress response in laparoscopic hysterectomy. Other clinical outcomes, including postoperative pain and nausea/vomiting, did not differ between the two groups.

Since the ANI monitor calculates heart rate variability mediated by parasympathetic changes, factors that can disturb the autonomic nervous system, such as pneumoperitoneum and Trendelenburg position in our study, may affect the values of ANI monitoring. CO_2_ pneumoperitoneum activates the sympathetic nervous system, which increases plasma catecholamine concentrations (23, 24). In previous studies based on heart rate variability (25–27), the ratio of low-frequency to high-frequency (LF/HF) band power increased after CO_2_ insufflation, which suggests sympathetic activation. While the baroreceptor reflex due to decreased cardiac output stimulates the sympathetic nervous system during pneumoperitoneum, increased venous return in the Trendelenburg position can reduce sympathetic overstimulation (27). In laparoscopic surgery in the Trendelenburg position, increased plasma levels of catecholamines, adrenocorticotropic hormone, and cortisol have been reported (28); however, the results of heart rate variability are conflicting (29–32). A previous study based on laparoscopic appendectomy or cholecystectomy demonstrated a decrease in ANI values after pneumoperitoneum inflation (33). Therefore, CO_2_ pneumoperitoneum and steep (30°) Trendelenburg position during surgery in our study can affect ANI values in addition to surgical nociception. In fact, we had more trouble maintaining adequate ANI values after the induction of pneumoperitoneum and Trendelenburg positioning than before surgery. Considering that the total duration of adequate ANIm values during surgery was not significantly different between groups despite the higher remifentanil administration in the ANI group, decreased parasympathetic activity (or increased sympathetic activity) by pneumoperitoneum may not be controlled well by opioid administration or may not be reflected by the ANI monitor.

Previous studies have reported that adequate nociception control guided by ANI monitoring or other nociception monitoring based on heart rate variability during surgery can reduce intraoperative opioid use and perioperative stress responses (34, 35). However, this study showed that ANI monitor-based nociception control during surgery did not reduce perioperative stress responses when compared with conventional nociception control. Furthermore, a higher dose of remifentanil was administered to the ANI group than to the control group to achieve adequate ANI values during surgery. In addition, heart rate and arterial blood pressure during anesthesia were significantly lower in the ANI group than in the control group, although more vasopressors were administered to avoid hypotension in the ANI group than in the control group. The low hemodynamic profiles in the ANI group may be due to the larger amount of remifentanil administered, which may be reflected in the profiles of perioperative stress responses between the two groups. Increased levels of catecholamines and glucocorticoid hormones have been reported in hypotensive situations (36, 37). Increased stress responses due to hypotension may mask the reduced stress responses following opioid administration.

The association between the autonomic nervous system and the immune system has been revealed. The vagus nerve, one of the main nerves of the parasympathetic nervous system, monitors and regulates peripheral inflammation (38). Therefore, we hypothesized that maintaining parasympathetic dominance during surgery by ANI monitoring could affect intra- and postoperative inflammatory responses, which may result in differences in tissue damage and inflammatory response during surgery between the two groups. However, there were no differences in inflammatory cytokines or markers of tissue damage (high mobility group box 1) between groups.

Postoperative pain outcomes after intraoperative ANI monitor-based nociception control can be affected by various factors, such as the type of surgery, anesthetic methods, or pain management strategy. However, previous studies have reported conflicting results. In contrast to favorable pain outcomes in spine and vascular surgeries (4, 11), ANI monitoring guidance did not improve postoperative pain in a variety of surgical patients (12–15) including laparoscopic surgery (16) and gynecologic surgery (17) (which included both laparoscopic and open abdominal surgeries). In the current study, although a higher dose of remifentanil was used in the ANI group than in the control group, the duration of the effect of remifentanil was short, and the dose of remifentanil in both groups was not high enough to induce hyperalgesia (39), postoperative pain outcomes were not different between the two groups. In addition, there was no difference between the two groups in the perioperative stress response or inflammatory response, which is consistent with the postoperative pain outcomes.

This study had some limitations. Firstly, the investigator in charge of anesthesia was not blinded to the randomization. However, the investigator in charge of laboratory measurements and postoperative outcome assessments was blinded to group allocation. Secondly, this study was conducted based on special clinical settings, such as laparoscopy in the steep Trendelenburg position in relatively young female patients, and the results of this study cannot be generalized to other patient populations. Thirdly, anticholinergic and ephedrine were used to treat bradycardia and hypotension, which can influence ANI monitoring. However, this medication can represent a clinical picture in this surgical setting.

In conclusion, the present study showed that nociception control with ANI monitoring according to the manufacturer’s recommendations did not improve perioperative stress response in terms of catecholamine and stress hormones in laparoscopic gynecologic surgery performed in the steep Trendelenburg position. In this clinical setting, despite the larger amount of opioid required to achieve adequate ANI values in the ANI group compared to the control group, ANI guidance could not improve postoperative clinical outcomes. When ANI monitoring is applied in laparoscopic surgeries in the Trendelenburg position, strategies other than maintaining the target range of ANIm may be required.

## Data availability statement

The raw data supporting the conclusions of this article will be made available by the authors, without undue reservation.

## Ethics statement

The studies involving human participants were reviewed and approved by the Institutional Review Board of Yonsei University Health System, Seoul, South Korea, approved this trial (#4-2020-0130) on April 1, 2020. The patients/participants provided their written informed consent to participate in this study.

## Author contributions

SK: acquisition, analysis, and interpretation of data, drafting and critical revision of the manuscript. CC, J-RL, SS, and YK: acquisition, analysis, interpretation of data, and critical revision of the manuscript. JL: study concept and design, acquisition, analysis, interpretation of data, drafting and critical revision of the manuscript. All authors read and approved the final manuscript.

## Funding

This research was supported by the Basic Science Research Program to JL through the National Research Foundation of Korea (NRF) funded by the Ministry of Science, ICT & Future Planning (NRF-2019R1C1C1005201).

## Conflict of interest

The authors declare that the research was conducted in the absence of any commercial or financial relationships that could be construed as a potential conflict of interest.

## Publisher’s note

All claims expressed in this article are solely those of the authors and do not necessarily represent those of their affiliated organizations, or those of the publisher, the editors and the reviewers. Any product that may be evaluated in this article, or claim that may be made by its manufacturer, is not guaranteed or endorsed by the publisher.
