# Supplementary material for: Effect of analgesia nociception index monitor-based nociception control on perioperative stress responses during laparoscopic surgery in Trendelenburg position: a randomized controlled trial
Source: Front Med (Lausanne). 2023 Aug 4;10:1196153. doi: 10.3389/fmed.2023.1196153 (PMC10436464; doi:10.3389/fmed.2023.1196153)
Supplement: Supplementary file 1 [file Table_1.DOCX]

Effect of analgesia nociception index monitoring on perioperative stress responses during laparoscopic surgery in Trendelenburg position: A randomized controlled trial

Seung Hyun Kim, Chul Ho Chang, Jeong Rim Lee, Seok Kyo Seo, Young In Kwon, Jae Hoon Lee*

*** Correspondence:** Jae Hoon Lee: NEOGENS@yuhs.ac

# Supplementary Figure 1


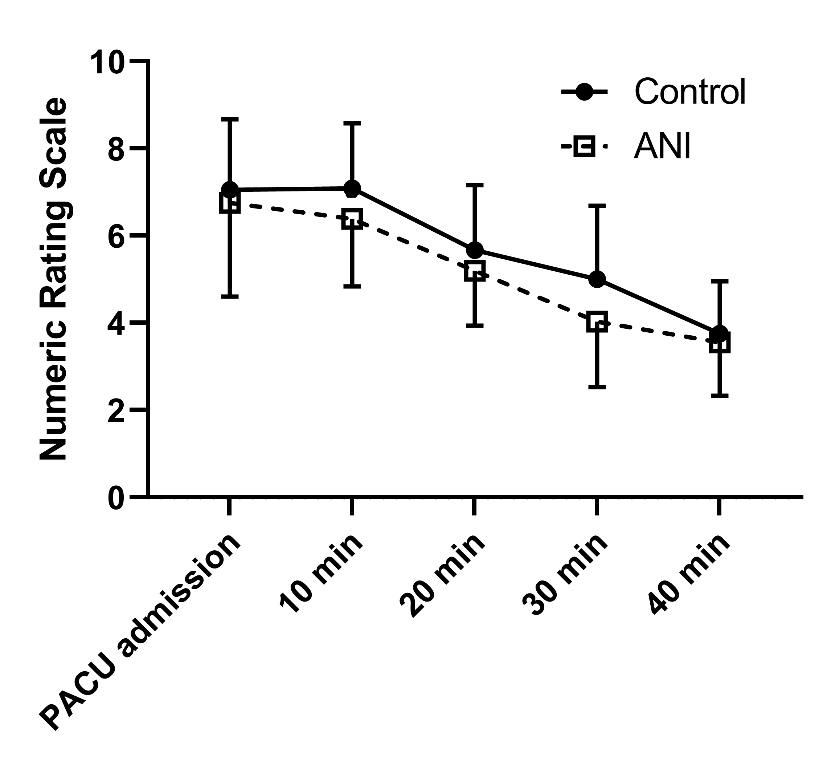


**Supplementary Figure 1.** Postoperative pain scores between the control and ANI group. ANI, analgesia nociception index.
